# Improving the influenza vaccination rate in patients visiting pediatric rheumatology clinics using automatic best practice alert in electronic patient records

**DOI:** 10.1186/1546-0096-10-S1-A106

**Published:** 2012-07-13

**Authors:** Anjali Patwardhan, Kelly Kelleher, Dennis Cunningham, Charles Spencer

**Affiliations:** 1 The Ohio State University and Nationwide Childrens Hospital, Columbus, OH, USA

## Purpose

Children with rheumatic disease who are infected with influenza have increased rates of complications. Influenza-related morbidity and mortality can be reduced by improving the flu vaccination rate. The purpose of the study is to look at the effectiveness of single information technology intervention in improving flu vaccination rate in children with rheumatic diseases.

## Methods

We examined three yearly cohorts (2007, 2008, and 2009) of rheumatology clinic patients from a large pediatric hospital for evidence of influenza vaccination in the electronic health record. We introduced an electronic health record (EHR) intervention automatic best practice alert reminder in our patients’ records from September 2009 until the end of the influenza season. We compared claims-based records of receipt over 3 years and conducted a Delphi survey of stakeholders after the flu season. In 2009, an EHR automatic best practice alert reminder to vaccinate patients was introduced. Using Clarity Report Write for EPIC, each chart was examined for evidence of influenza vaccination in order to test for vaccination rate difference amongst the cohorts. We employed logistic regression equations to control for possible confounders (age, sex, ethnicity, insurance status, distance from clinic and attending physician) using SAS 9.1.3. We conducted qualitative interviews with participating clinicians to assess their perspectives on the addition of the reminder.

## Results

There was a significant difference in the probability of being vaccinated before and after intervention (p value <0.0001).With the rate increased from 5.9 % in 2007 and 7.8% in 2008 to 25.5 % in 2009. All three years, individual attending’s contribution and ethnicity of patients had significant effects on vaccination rate. Confounders such as age, sex, insurance status and distance from clinic had no effect on the vaccination rate.

**Figure 1:**
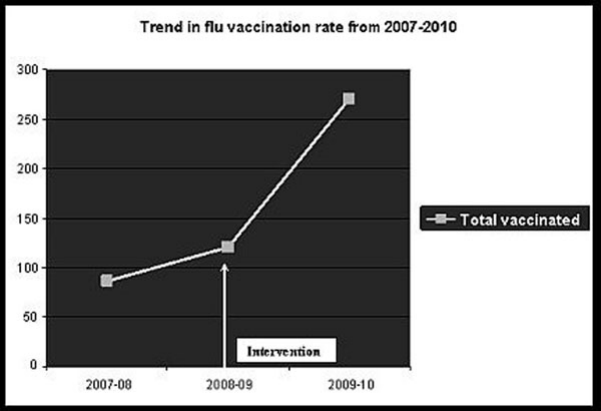


## Conclusion

EHR-embedded information in past studies has been only modestly effective in improving care for many chronic conditions. Our automatic best practice alert reminder for flu-vaccine appears to be effective for changing behaviors and improving the vaccination rate in rheumatology clinics.

## Disclosure

Anjali Patwardhan: None; Kelly Kelleher: None; Dennis Cunningham: None; Charles Spencer: None.

